# Overexpression of diglucosyldiacylglycerol synthase leads to daptomycin resistance in *Bacillus subtilis*

**DOI:** 10.1128/jb.00307-24

**Published:** 2024-09-05

**Authors:** Ryogo Yamamoto, Kazuya Ishikawa, Yusuke Miyoshi, Kazuyuki Furuta, Shin-Ichi Miyoshi, Chikara Kaito

**Affiliations:** 1 Graduate School of Medicine, Dentistry and Pharmaceutical Sciences, Okayama University, Okayama, Japan; 2 Research Center for Intestinal Health Science, Okayama University, Okayama, Japan; University of Illinois Chicago, Chicago, Illinois, USA

**Keywords:** diglucosyldiacylglycerol, daptomycin, phospholipid

## Abstract

The lipopeptide antibiotic daptomycin exhibits bactericidal activity against Gram-positive bacteria by forming a complex with phosphatidylglycerol (PG) and lipid II in the cell membrane, causing membrane perforation. With the emergence of daptomycin-resistant bacteria, understanding the mechanisms of bacterial resistance to daptomycin has gained great importance. In this study, we aimed to identify the genetic factors contributing to daptomycin resistance in *Bacillus subtilis*, a model Gram-positive bacterium. Our findings demonstrated that overexpression of *ugtP*, which encodes diglucosyldiacylglycerol synthase, induces daptomycin resistance in *B. subtilis*. Specifically, overexpression of *ugtP* resulted in increased levels of diglucosyldiacylglycerol (Glc_2_DAG) and decreased levels of acidic phospholipids cardiolipin and PG, as well as the basic phospholipid lysylphosphatidylglycerol. However, *ugtP* overexpression did not alter the cell surface charge and the susceptibility to the cationic antimicrobial peptide nisin or the cationic surfactant hexadecyltrimethylammonium bromide. Furthermore, by serial passaging in the presence of daptomycin, we obtained daptomycin-resistant mutants carrying *ugtP* mutations. These mutants showed increased levels of Glc_2_DAG and a >4-fold increase in the minimum inhibitory concentration of daptomycin. These results suggest that increased Glc_2_DAG levels, driven by *ugtP* overexpression, modify the phospholipid composition and confer daptomycin resistance in *B. subtilis* without altering the cell surface charge of the bacteria.

**IMPORTANCE:**

Daptomycin is one of the last-resort drugs for the treatment of methicillin-resistant *Staphylococcus aureus* infections, and the emergence of daptomycin-resistant bacteria has become a major concern. Understanding the mechanism of daptomycin resistance is important for establishing clinical countermeasures against daptomycin-resistant bacteria. In the present study, we found that overexpression of *ugtP*, which encodes diglucosyldiacylglycerol synthase, induces daptomycin resistance in *B. subtilis*, a model Gram-positive bacteria. The overexpression of *UgtP* increased diglucosyldiacylglycerol levels, resulting in altered phospholipid composition and daptomycin resistance. These findings are important for establishing clinical strategies against daptomycin-resistant bacteria, including their detection and management.

## INTRODUCTION

The lipopeptide antibiotic daptomycin is used to treat severe infections such as infective endocarditis and deep-skin infections caused by methicillin-resistant *Staphylococcus aureus* (MRSA). Daptomycin exerts its bactericidal effect by forming a complex with phosphatidylglycerol (PG) and lipid II in the bacterial cell membrane, a process that is dependent on calcium ions (Ca^2+^). This complex formation leads to membrane perforation in Gram-positive bacteria (1). With the increasing emergence of daptomycin-resistant Gram-positive bacteria, understanding the mechanisms of daptomycin resistance has become crucial. In *S. aureus*, mutations in the *mprF* gene, which is responsible for the lysylation of PG, are known to confer daptomycin resistance, sometimes without affecting the bacterial cell surface charge (2–6). In *Enterococcus faecalis*, daptomycin resistance is associated with mutations in *liaF*, which plays a role in the stress response of the cell membrane, or in *gdpD*, which is involved in phospholipid metabolism (7). Similarly, in *Bacillus subtilis*, mutations in the *pgsA* gene, which is responsible for PG synthesis, lead to daptomycin resistance by decreasing the level of PG produced (8).

UgtP is an enzyme responsible for synthesizing diglucosyldiacylglycerol (Glc_2_DAG), a glyceroglycolipid, from UDP-glucose and diacylglycerol (DAG) (9). Glc_2_DAG forms a lamellar phase in the cell membrane, while monoglucosyldiacylglycerol forms hexagonal or cubic phases (10–12). Glc2DAG can be converted into glycerophospho-diglucosyldiacylglycerol (GP-DGDAG) through its reaction with PG (13). GP-DGDAG acts as an anchor for lipoteichoic acid (LTA) in Gram-positive bacteria (14, 15). The *ugtP* gene influences various bacterial phenotypes. In *Bacillus subtilis*, the *ugtP*-deletion mutant fails to produce Glc_2_DAG, has elongated LTA, exhibits reduced swarming activity, and shows increased expression of sigma factors that respond to environmental changes (16–18). In *Staphylococcus aureus*, a deletion mutant of *ypfP*, which is a homolog of *ugtP*, demonstrates reduced LTA levels (19), increased autolysis (19, 20), decreased colony spreading (21), and reduced secretion of leukocidin (LukAB) (22). Additionally, in invasive Group A Streptococcus, Glc2DAG plays a role in evading immune cell attacks (23).

The *ugtP* gene is also implicated in susceptibility to various antimicrobial agents. For instance, *Bacillus subtilis ugtP*-deletion mutants exhibit increased susceptibility to sublancin, a cationic antimicrobial peptide (17). Additionally, MRSA strains with *ugtP* deletions show altered cell size and cell wall integrity, making them more susceptible to β-lactam antibiotics and cell wall hydrolases (24). However, the relationship between *ugtP* and daptomycin resistance has not been previously explored. In the present study, we aimed to identify the genetic factors contributing to daptomycin resistance in *B. subtilis*, a model Gram-positive bacterium. We demonstrated that overexpression of *ugtP* in *B. subtilis* leads to daptomycin resistance.

## RESULTS

### Overexpression of Glc_2_DAG synthase in *B. subtilis* leads to daptomycin resistance

**Fig 1:**
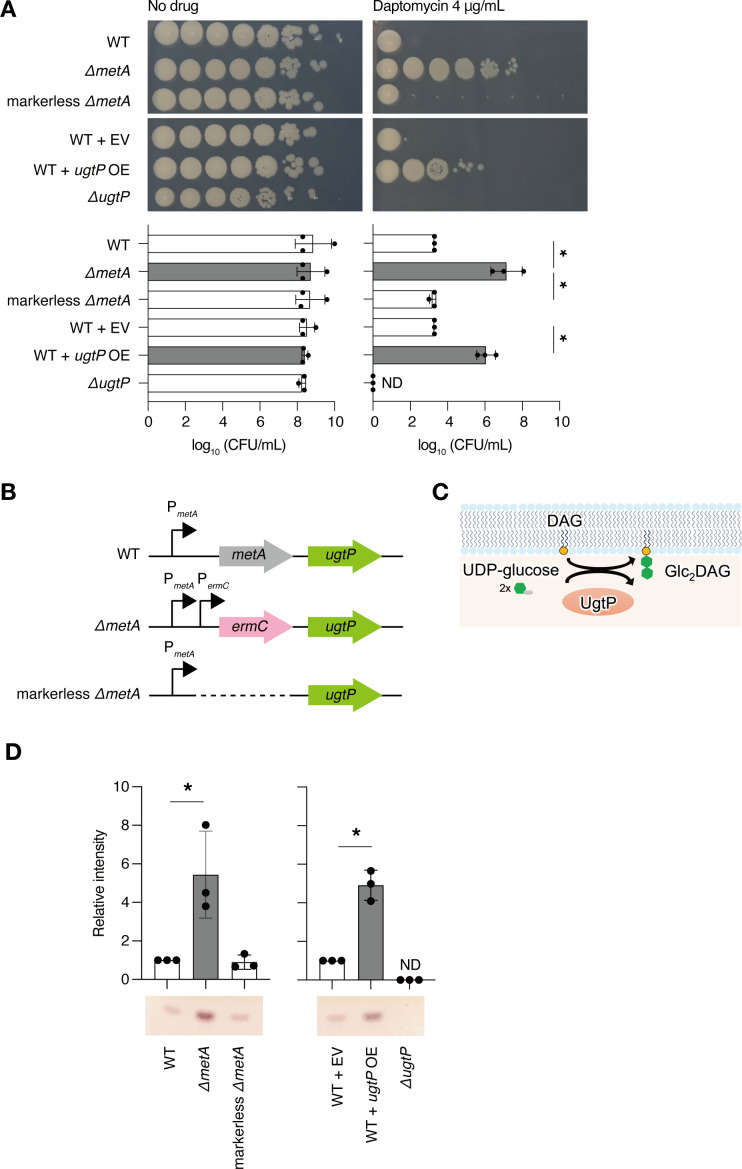
Increased levels of Glc_2_DAG in *Bacillus subtilis* led to daptomycin resistance. (A) Overnight cultures of the wild-type (WT) strain, the *metA*-deletion mutant (Δ*metA*), the markerless *metA*-deletion mutant (markerless Δ*metA*), the wild-type strain transformed with empty vector (WT + EV), the wild-type strain transformed with *ugtP*-overexpressing plasmid (WT + *ugtP* OE), and the *ugtP*-deletion mutant strain (Δ*ugtP*) were serially diluted 10-fold, spotted onto Luria-Bertani agar plates containing 1-mM isopropyl-beta-d-thiogalactopyranosid supplemented with or without daptomycin 4 µg/mL, and incubated at 37°C overnight. The log_10_ CFU/mL value was calculated based on the number of colonies formed (graph). Data are presented as mean ± SD from three independent experiments. Statistical differences between the groups were analyzed using Tukey’s multiple-comparison test. *P < 0.05. (B) Schematic representation of the genome locus encompassing the *metA* and the *ugtP* genes in the WT, Δ*metA*, and markerless Δ*metA* strains. P*_metA_* and P*_ermC_* represent the promoter regions of the *metA* and erythromycin resistance cassette (*ermC*) genes, respectively. (C) Schematic representation of diglucosyldiacylglycerol (Glc_2_DAG) synthesis in *B. subtilis*. In *B. subtilis*, UgtP synthesizes Glc_2_DAG from UDP-glucose and diacylglycerol (DAG). (D) Total lipids of bacterial strains in A were extracted and analyzed using thin-layer chromatography. The signal intensity of Glc_2_DAG was measured. Data are expressed as mean ± SD from three independent experiments. Levels relative to the WT (left graph) and WT + EV (right graph) are shown. Statistical differences between groups were analyzed using Student’s *t*-test. *P < 0.05.

Using the *B. subtilis* gene knockout library (25), we searched for a gene knockout strain that grew on Luria-Bertani (LB) agar plates containing 4 µg/mL of daptomycin. The deletion mutant of *metA*, which encodes succinyl-O-methyltransferase showed resistance to daptomycin (Fig. 1A). In the absence of daptomycin, the *metA*-deletion mutant showed colony formation comparable to that of the wild-type strain on LB agar plates but showed slightly delayed growth in liquid LB (Fig. 1A; Fig. S1). To confirm that the lack of *metA* leads to daptomycin resistance, we performed a complementation experiment. *metA* was introduced into the *amyE* locus of *B. subtilis* to construct a *metA*-complemented strain. Introducing *metA* into the *metA*-deletion mutant did not alter daptomycin sensitivity (Fig. S2). Because *ugtP* is located downstream of the *metA* locus (Fig. 1B), we hypothesized that the daptomycin resistance of the *metA*-deletion mutant was due to the polar effect of the erythromycin resistance cassette (*ermC*), an antibiotic resistance marker, in the *metA* locus. Therefore, we constructed a *metA* markerless deletion mutant by removing *ermC* from the *metA*-deletion mutant (Fig. 1B). The *metA* markerless deletion mutant did not show daptomycin resistance (Fig. 1A). Furthermore, overexpression of *ugtP* in the wild-type strain using an isopropyl-beta-d-thiogalactopyranosid (IPTG)-inducible promoter resulted in daptomycin resistance (Fig. 1A). In the absence of daptomycin, the *ugtP*-overexpressed strain showed colony formation and growth comparable to that of the vector-transformed strain (Fig. 1A; Fig. S1). These results suggest that increased expression of *ugtP* leads to daptomycin resistance.

Next, total lipids were extracted from the bacteria and examined using thin-layer chromatography (TLC) to investigate whether overexpression of *ugtP*, which encodes Glc_2_DAG synthase (Fig. 1C), increased the levels of Glc_2_DAG. The analysis revealed that the Glc_2_DAG levels in the *ugtP*-overexpressed strain and *metA*-deletion mutant were greater than those in the vector-transformed and wild-type strains (Fig. 1D). The level of Glc_2_DAG was not altered in the *metA* markerless deletion mutant in comparison with that in the wild-type strain (Fig. 1D). The *ugtP*-deletion mutant did not produce Glc_2_DAG (Fig. 1D) and was more susceptible to daptomycin than the wild-type strain (Fig. 1A). Since daptomycin resistance correlates with Glc_2_DAG accumulation, these results suggest that Glc_2_DAG synthesized by *ugtP* is involved in daptomycin resistance.

### Overexpression of Glc_2_DAG synthase results in decreased bactericidal activity of daptomycin

**Fig 2:**
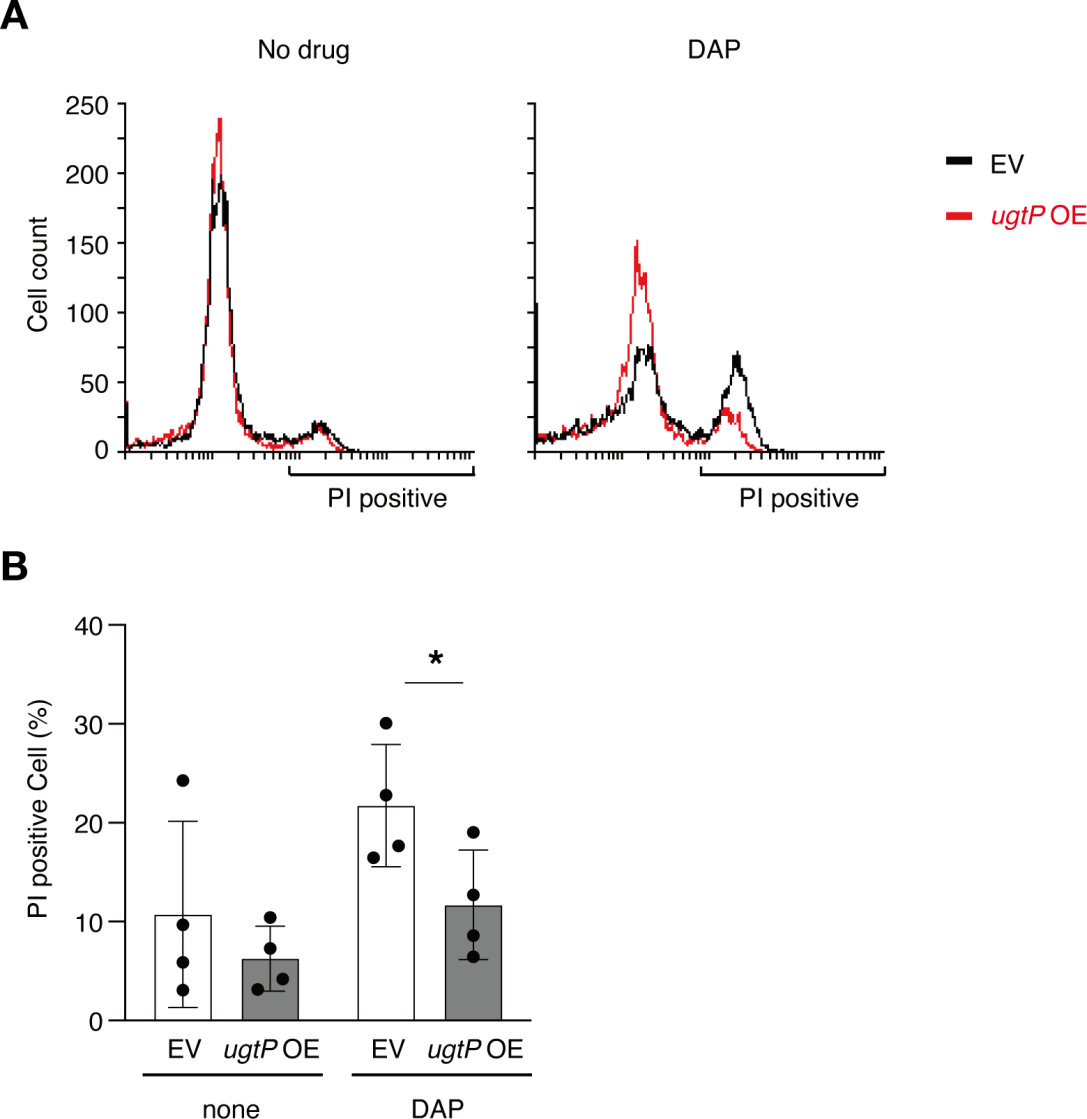
UgtP overexpression leads to the reduced bactericidal ability of daptomycin (DAP). (A) Logarithmically growing cells of the wild-type strain transformed with empty vector (EV) and the wild-type strain transformed with *ugtP*-overexpressing plasmid (*ugtP* OE) were stained by propidium iodide (PI) after treatment with or without daptomycin 10 µg/mL for 5 min at 37°C. The vertical axis represents the number of cells, and the horizontal axis represents the fluorescence intensity of PI. (B) The percentage of PI-positive cells was measured in panel A. Data are expressed as mean ± SD from three independent experiments. The statistical significance of the differences was analyzed using Student’s *t*-test. *P < 0.05.

To analyze the effect of Glc_2_DAG on daptomycin activity in *B. subtilis* in more detail, we measured the number of dead bacteria stained with propidium iodide (PI) using flow cytometry. Bacterial cells with holes in their membranes due to daptomycin activity were speculated to be stained with PI. After daptomycin exposure, the percentage of PI-positive cells in the *ugtP*-overexpressed strain was half that in the vector-transformed strain (Fig. 2A and B). These results suggest that the bactericidal activity of daptomycin was reduced in the *ugtP*-overexpressed strain.

### Overexpression of Glc_2_DAG synthase did not change the bacterial cell surface charge

**Fig 3:**
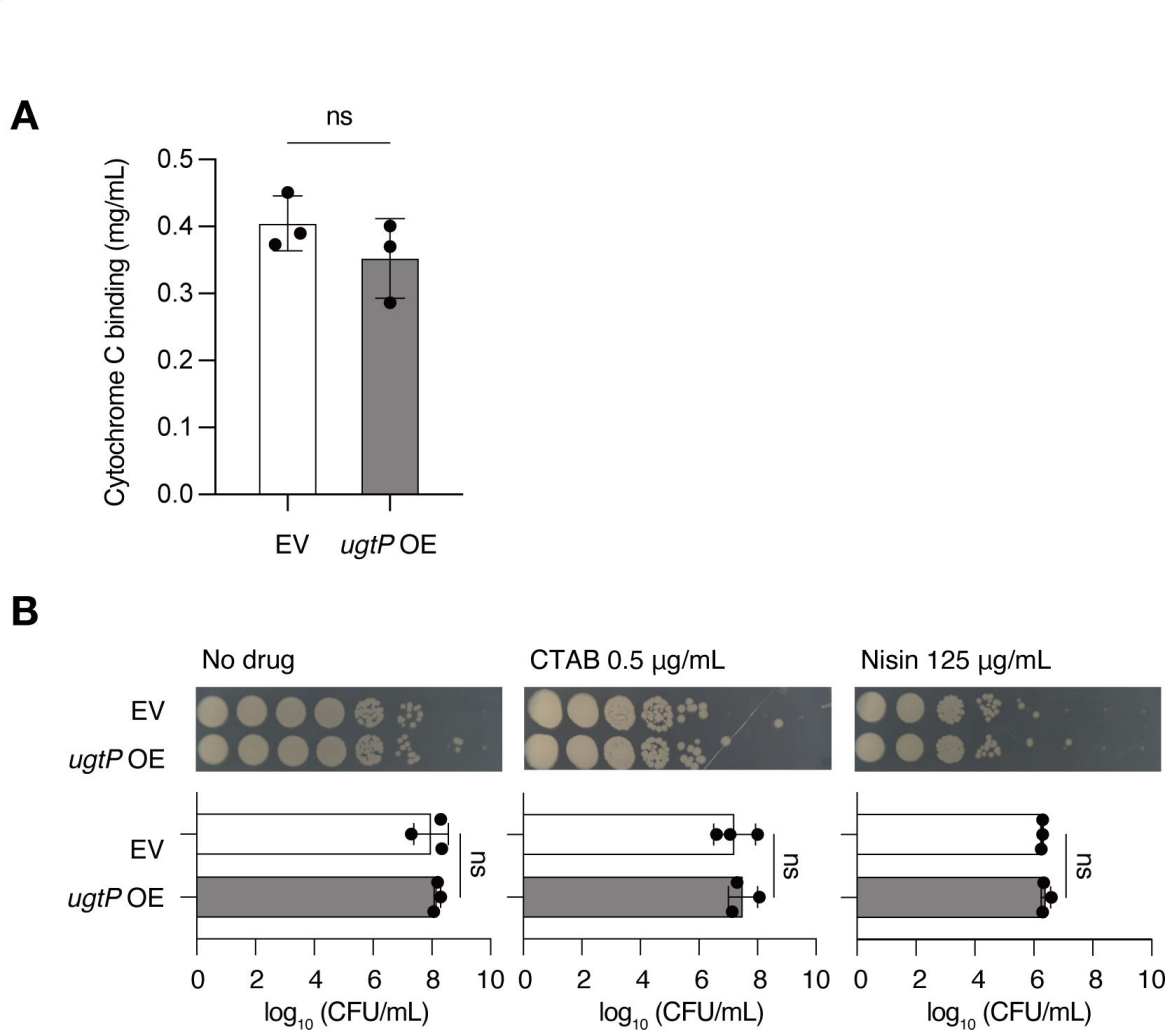
The *ugtP*-overexpressed strain does not show surface charge alterations. (A) Surface charge of *B. subtilis* was evaluated by cytochrome C binding. Overnight cultures of the wild-type strain transformed with an empty vector (EV) and the wild-type strain transformed with *ugtP*-overexpressing plasmid (*ugtP* OE) were subjected to a cytochrome c binding assay. Data are presented as mean ± SD from three independent experiments. Statistical differences between groups were analyzed using Student’s *t*-test. (B) Overnight cultures of the wild-type strain transformed with EV and the wild-type strain transformed with *ugtP* OE were serially diluted 10-fold, spotted on Luria-Bertani agar plates supplemented with or without 0.5 µg/mL of hexadecyltrimethylammonium bromide or 125 µg/mL of nisin, and incubated at 37°C overnight. Log_10_ CFU/mL was calculated from the number of colonies formed (graph below). Data are presented as mean ± SD from three independent experiments. The statistical significance of differences between groups was analyzed using Student’s *t*-test. ns, not significant.

Based on a study reporting that the *mprF-*overexpressed *B. subtilis* strain shows daptomycin resistance along with a decrease in the negative charge of the cell membrane (26), we examined whether the *ugtP*-overexpressed strain also showed an altered cell surface charge by using the binding assay of cytochrome c, which binds to the negatively charged cell surface. No significant difference in cytochrome c binding was observed between the vector-transfected and *ugtP-*overexpressed strains (Fig. 3A). Furthermore, the *ugtP-*overexpressed strain and the vector-transformed strain showed similar sensitivity to the cationic antimicrobial agents, nisin and hexadecyltrimethylammonium bromide (CTAB) (Fig. 3B). These results suggest that the daptomycin resistance caused by *ugtP* overexpression is not attributable to alterations in the cell surface charge.

### Overexpression of Glc_2_DAG synthase alters the phospholipid composition of the cell membrane

**Fig 4:**
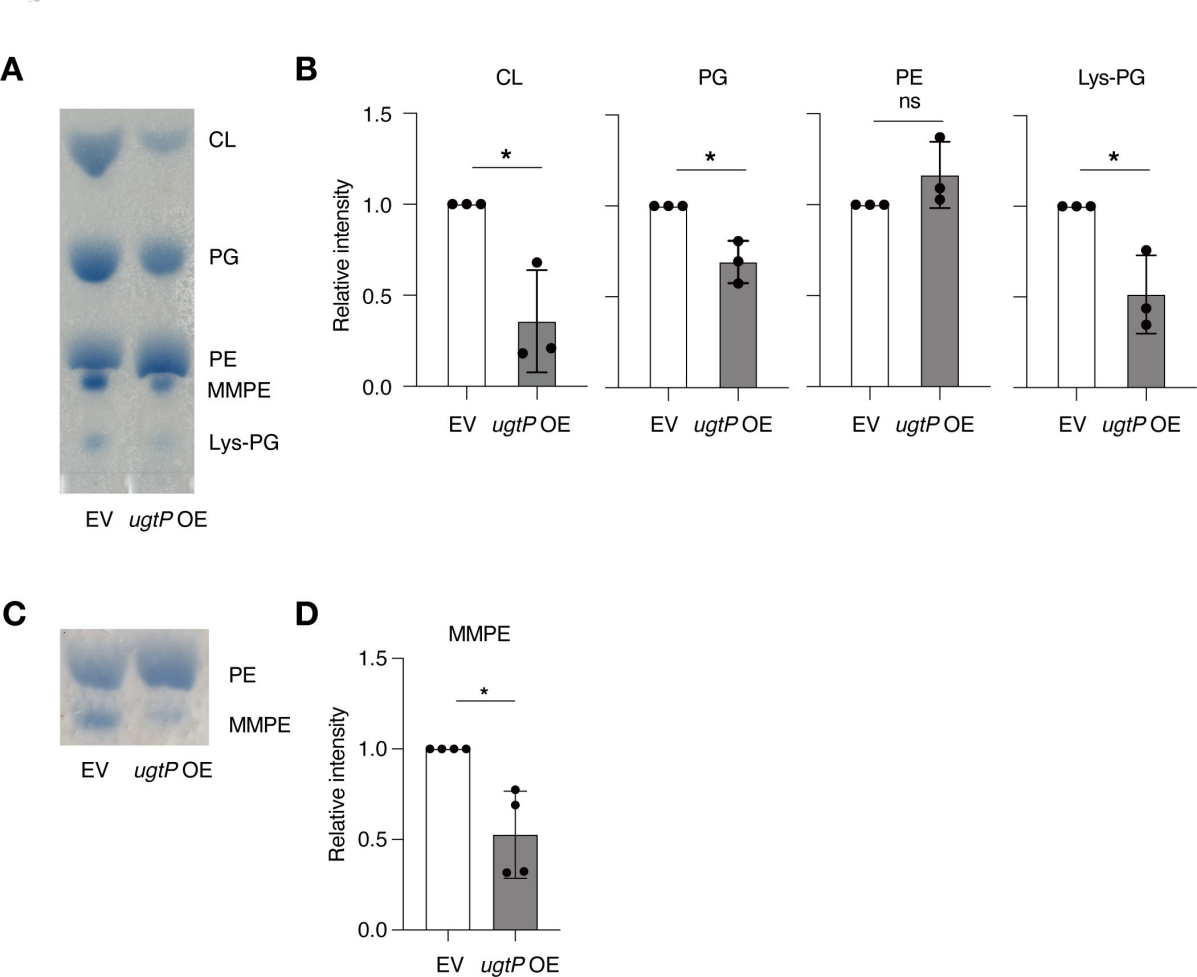
UgtP overexpression alters phospholipid composition. (A) Total lipids were extracted from overnight cultures of the wild-type strain transformed with an empty vector (EV) and the wild-type strain transformed with *ugtP*-overexpressing plasmid (*ugtP* OE) and analyzed by TLC. (B) Signal intensities of CL, PG, PE, and Lys-PG were measured in panel A. Relative levels of phospholipids against those in the EV strain are shown. Data are shown as mean ± SD from three independent experiments. The statistical significance of differences between groups was analyzed using Student’s *t*-test. *P < 0.05, significant difference. (C) Total lipids were extracted from overnight cultures of the wild-type strain transformed with an empty vector (EV) and the wild-type strain transformed with *ugtP*-overexpressing plasmid (*ugtP* OE) and subjected to TLC analysis for PE and monomethyl-phosphatidylethanolamine (MMPE). (D) Signal intensities of MMPE were measured in panel C. Relative levels of MMPE against that in the EV strain are shown. Data are shown as mean ± SD from four independent experiments. The statistical significance of differences between groups was analyzed using Student’s *t*-test. *P < 0.05, significant difference. CL, cardiolipin; Lys-PG, lysylphosphatidylglycerol; ns, not significant; PE, phosphatidylethanolamine; PG, phosphatidylglycerol.

Decreased levels of PG lead to daptomycin resistance in *B. subtilis* and *S. aureus* (8, 27). Therefore, we compared the levels of phospholipids in the vector-transformed and *ugtP*-overexpressed strains by TLC. The levels of the major phospholipids of *B. subtilis*, namely, cardiolipin (CL), PG, and lysylphosphatidylglycerol (Lys-PG), were determined by comparing the Rf values with those reported previously (28). Compared to the vector-transformed strain, the *ugtP*-overexpressed strain showed lower levels of CL, PG, and Lys-PG (Fig. 4A and B). The level of phosphatidylethanolamine (PE) was not significantly different between the *ugtP*-overexpressed and vector-transformed strains (Fig. 4A and B). The spot with a lower Rf value than PE was identified as monomethyl-phosphatidylethanolamine (MMPE), a methylated PE (29). The *ugtP*-overexpressed strain had a reduced level of MMPE compared to the vector-transformed strain (Fig. 4C and D). These results suggest that *ugtP* overexpression decreases the levels of CL, PG, Lys-PG, and MMPE.

### Overexpression of Glc_2_DAG synthase increases the level of lipoteichoic acid

**Fig 5:**
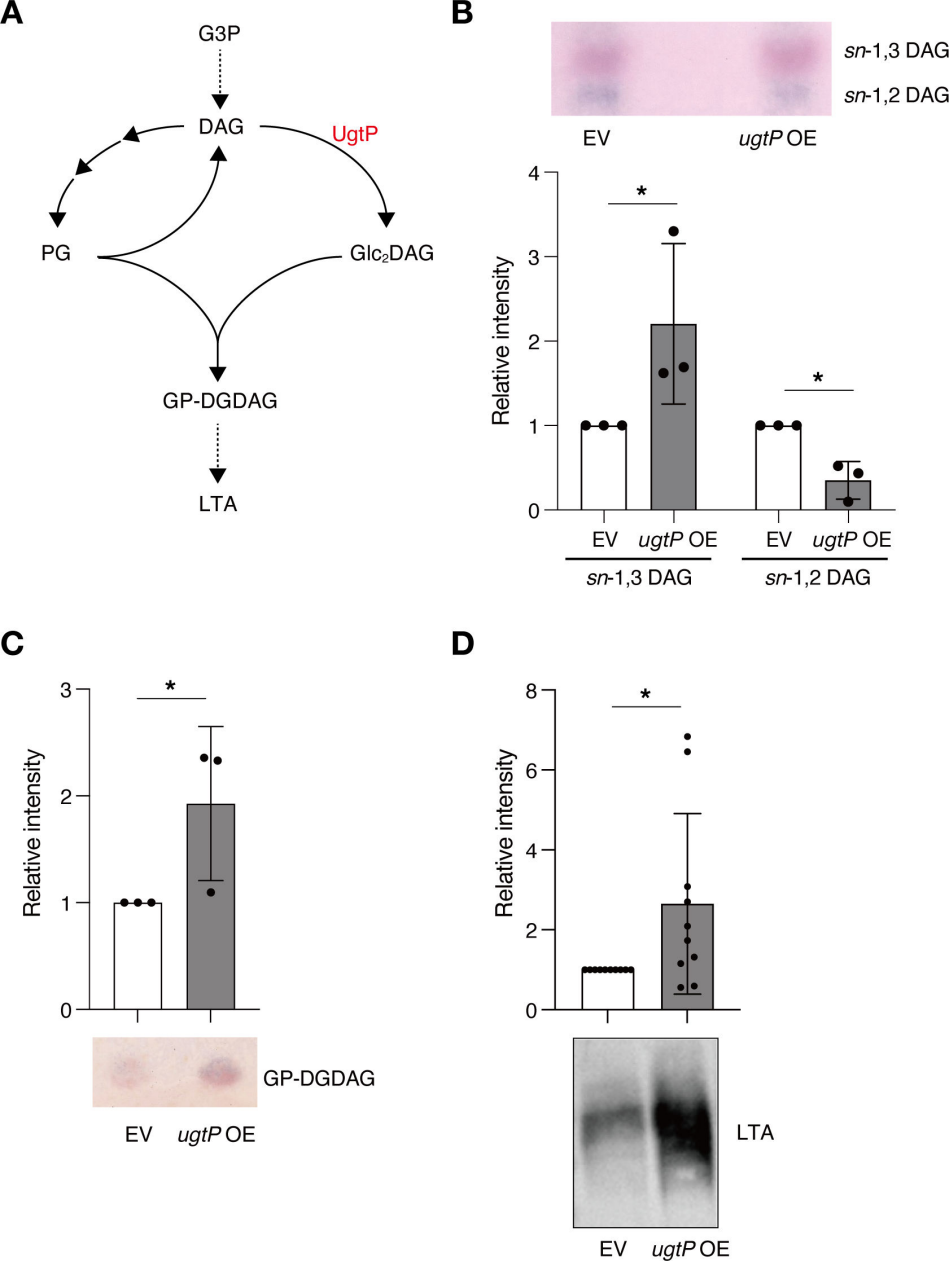
Overexpression of Glc_2_DAG synthase increases the level of lipoteichoic acid. (A) Lipoteichoic synthetic pathway. DAG is produced from G3P (glycerol-3-phosphate). PG made from DAG reacts with Glc_2_DAG to form GP-DGDAG, which changes to LTA. (B) Total lipids of bacterial strains were extracted and analyzed using TLC. Relative levels of *sn*-1,3 DAG and *sn*-1,2 DAG against those in the EV strain are shown. Data are expressed as mean ± SD from three independent experiments. The statistical significance of differences between groups was analyzed using Student’s *t*-test. *P < 0.05. (C) Total lipids of bacterial strains were extracted and were subjected to TLC analysis for GP-DGDAG. The relative level of GP-DGDAG against that in the EV strain is shown. Data are expressed as mean ± SD from three independent experiments. Statistical differences between groups were analyzed using Student’s *t*-test. *P < 0.05. (D) The wild-type strain transformed with an empty vector (EV) and the wild-type strain transformed with *ugtP*-overexpressing plasmid (*ugtP* OE) were cultured overnight, and the lipoteichoic acids were extracted. Lipoteichoic acids were detected by Western blotting using an anti-lipoteichoic acid antibody. The relative level of LTA against that in the EV strain is shown. Data are presented as mean ± SD from 10 independent experiments. The statistical significance of differences between groups was analyzed using Student’s *t*-test. *P < 0.05.

As shown in Fig. 5A, the decreased level of PG and the increased production of Glc_2_DAG could alter the level of DAG in the *ugtP*-overexpressed strain. Therefore, we measured DAG levels in the *ugtP-*overexpressed strain. Consistent with the previous studies (30–32), we identified two DAGs, *sn*-1,3 DAG and *sn*-1,2 DAG, with different Rf values (Fig. 5B). The *ugtP*-overexpressed strain had a higher level of *sn*-1,3 DAG and a lower level of *sn*-1,2 DAG than the vector-transformed strain (Fig. 5B). These results suggest that *ugtP* overexpression alters the levels of DAGs.

Glc_2_DAG produced by UgtP reacts with PG to form GP-DGDAG, which repeatedly incorporates glycerol phosphate moiety from PG to become LTA (13–15; Fig. 5A); therefore, we measured the levels of GP-DGDAG and LTA. The *ugtP*-overexpressed strain had higher levels of GP-DGDAG and LTA than the vector-transformed strain (Fig. 5C and D). These results suggest that increased production of Glc_2_DAG in the *ugtP*-overexpressed strain leads to upregulation of the LTA synthesis pathway.

### Emergence of daptomycin-resistant strains producing a large level of Glc_2_DAG in an experimental evolution

**Fig 6:**
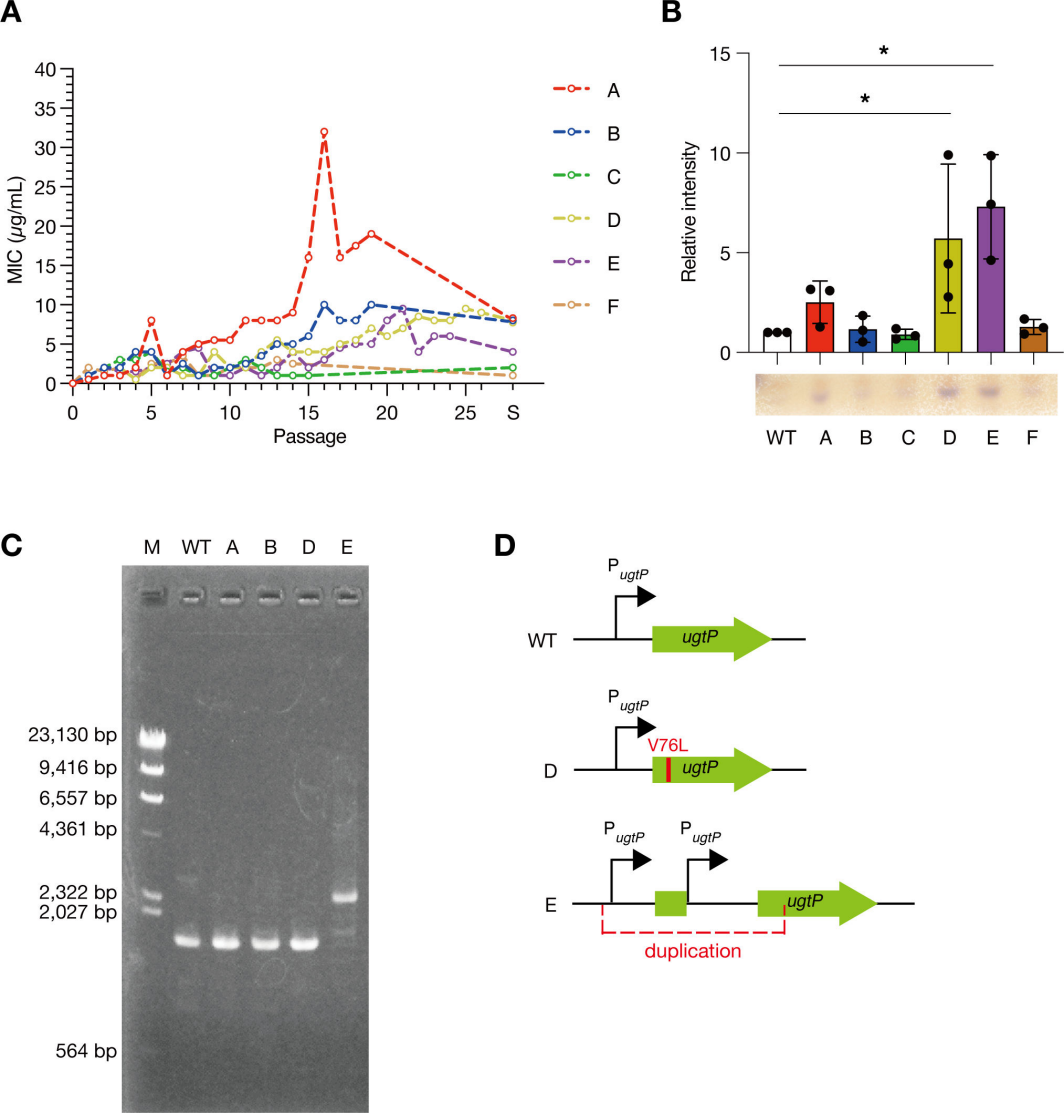
Daptomycin-resistant strains isolated in an experimental evolution have *ugtP* mutations and increased levels of diglucosyldiacylglycerol. (A) Six lineages of *B. subtilis* (A–F) were serially passaged in a daptomycin-containing medium and their MICs were measured. The horizontal axis represents the number of passages, and the vertical axis represents the daptomycin MIC. Finally, a single colony was isolated from the passaged cultures, named strains A–F, for which the MICs are shown at S on the horizontal axis. (B) Total lipid content was extracted from six strains (A–F), and the level of diglucosyldiacylglycerol was measured by thin-layer chromatography. Data are shown as mean ± SD from three independent experiments. The relative levels of diglucosyldiacylglycerol to those in the wild-type strain (WT) are shown. The significance of differences between groups was analyzed using Dunnett’s multiple-comparison test. *P < 0.05. (C) The *ugtP* region was amplified by PCR and subjected to DNA electrophoresis. (D) Schematic representation of the *ugtP* locus in the wild-type, D, and E strains.

To investigate whether mutants with increased Glc_2_DAG accumulation emerged after daptomycin exposure, we performed serial passaging in the presence of daptomycin. Six lineages were independently cultured for 15–26 passages in the presence of daptomycin, and four strains (A, B, D, and E) with more than fourfold higher minimum inhibitory concentration (MIC) than that of the wild-type strain were obtained (Fig. 6A). TLC analysis revealed that the level of Glc_2_DAG increased in two of the four daptomycin-resistant strains (Fig. 6B). To determine whether these daptomycin-resistant mutants carried mutations in *ugtP*, the *ugtP* locus was PCR amplified and sequenced. In strain E, the PCR product was larger than that in the wild-type strain (Fig. 6C), and sequence analysis revealed that a partial region encompassing the *ugtP* promoter region was duplicated (Fig. 6D). In strain D, guanine at position 226 was replaced by cytosine, resulting in the exchange of valine-76 of UgtP for leucine (Fig. 6D). In the other two daptomycin-resistant strains, which did not show an increase in the level of Glc_2_DAG, no mutations were observed in *ugtP*. These results indicated that daptomycin exposure results in an increase in the level of Glc_2_DAG in daptomycin-resistant strains, which is caused by *ugtP* mutations.

## DISCUSSION

In this study, *ugtP* overexpression led to daptomycin resistance in *B. subtilis*. To our knowledge, this is the first report describing the relationship between increased levels of Glc*_2_*DAG and daptomycin resistance in *B. subtilis*.

**Fig 7:**
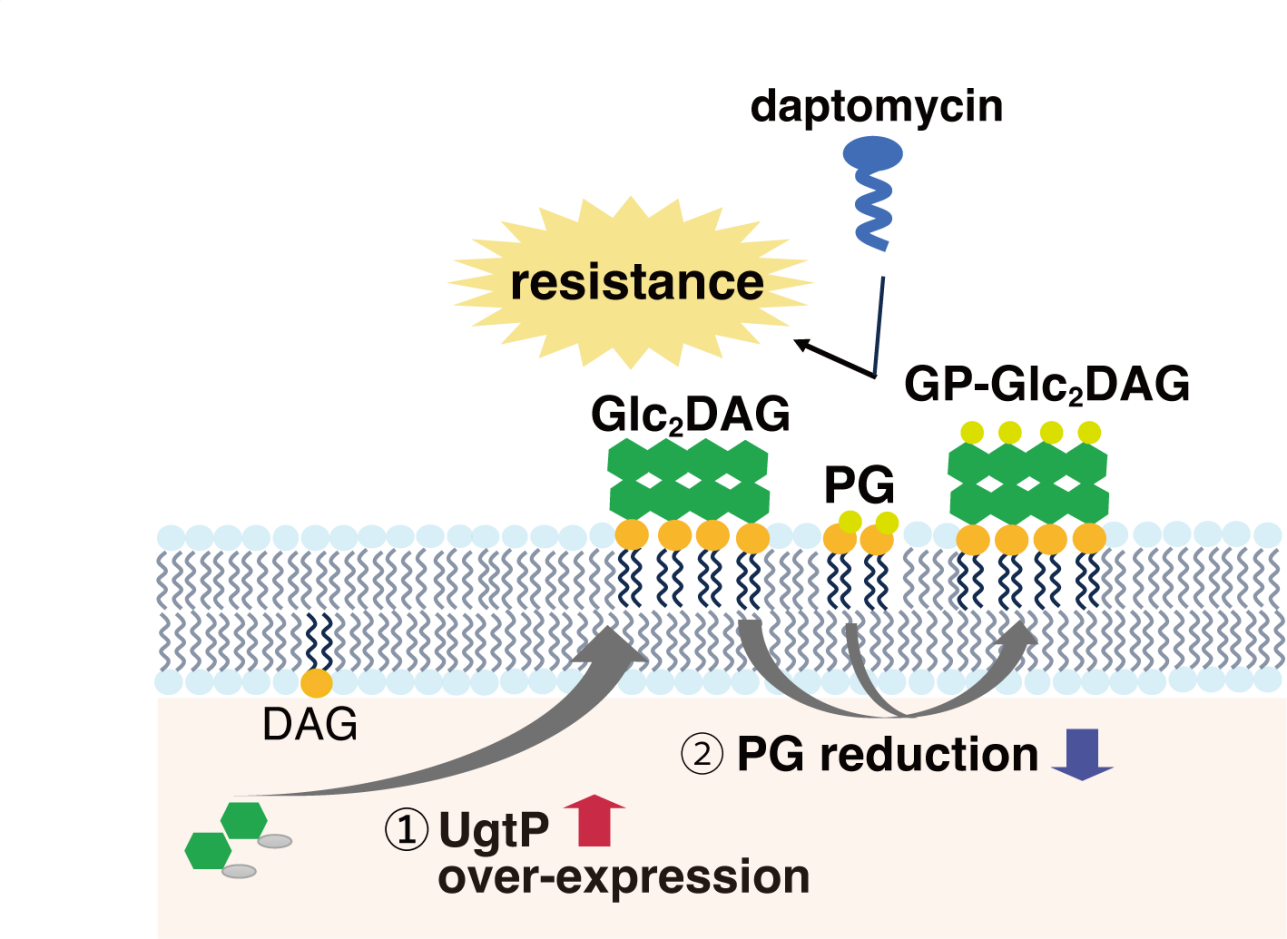
Model in which *ugtP* overexpression leads to daptomycin resistance. UgtP overexpression increases diglucosyldiacylglycerol levels (1) and decreases phosphatidylglycerol (PG) levels (2). A decrease in PG levels may decrease the efficiency of the daptomycin-PG complex formation.

Overexpression of *ugtP* in *B. subtilis* increased the level of Glc_2_DAG and altered the level of phospholipids (Fig. 1 and 4). Because Glc_2_DAG reacts with the phosphoglycerol group of PG to form GP-DGDAG (33) (Fig. 5A), the increased Glc_2_DAG levels in the *ugtP*-overexpressed strain would lead to a decrease in the amount of PG (Fig. 7), possibly leading to a consequent reduction in the efficiency of complex formation between daptomycin and PG (Fig. 7). Eventually, the impaired formation of the daptomycin-PG complex could have contributed to daptomycin resistance as daptomycin forms a complex with PG and causes membrane perforation (1). In addition to PG, CL and Lys-PG levels also decreased in the *ugtP*-overexpressed strain. As CL is a dimer of PG (34) and Lys-PG is a modified PG, reductions in these phospholipids may also contribute to daptomycin resistance. While the proportion of Glc_2_DAG to total lipids in exponentially growing *B. subtilis* cells has been reported to be approximately 10% (35), the present study revealed that the *ugtP*-overexpressed strain produced fivefold more Glc_2_DAG than the vector-transformed strain (Fig. 1). Thus, the *ugtP*-overexpressed strain presumably produces a large amount of Glc_2_DAG in the total lipid content, which may directly affect the action of daptomycin. Further investigation is needed to examine the effects of changes in lipid composition on daptomycin activity.

Our findings showed that the *ugtP*-overexpressed strain had increased levels of LTA (Fig. 5). This observation is noteworthy in light of conflicting evidence in the literature. A previous study identified LTA as a target of daptomycin (36). However, other research has suggested that LTA is not a target of daptomycin in *S. aureus* and enterococci (37). Given these discrepancies, the role of LTA in daptomycin resistance in *B. subtilis* remains unclear. Future investigations should address this issue by evaluating the daptomycin resistance profile of an LTA-deficient mutant to determine whether LTA contributes to the observed resistance mechanism in *B. subtilis*.

Daptomycin resistance in *S. aureus* is not fully explained by electric repulsion between the bacterial cell surface and daptomycin (27). In the present study, the cell surface charge of the *B. subtilis* strain overexpressing *ugtP* was unchanged despite a reduction in the levels of acidic phospholipids CL and PG (Fig. 3 and 4). The decrease in the basic phospholipid Lys-PG levels may have counterbalanced the effects of the reduced acidic phospholipids in the *ugtP*-overexpressed strain. Furthermore, the *ugtP*-overexpressed strain showed similar sensitivity to cationic antimicrobial substances such as nisin and CTAB compared to the vector-transformed strain. Therefore, the daptomycin resistance of the *ugtP-*overexpressed *B. subtilis* strain cannot be attributed to the alterations in the cell surface charge.

This study also demonstrated elevated Glc_2_DAG levels and daptomycin resistance in *B. subtilis* strains serially passaged in a daptomycin-containing medium. Sequencing analysis identified two independent spontaneous mutations in *ugtP*: one involving a duplication of the *ugtP* promoter and the other an amino acid substitution. These mutations are likely to enhance *ugtP* transcription and UgtP activity/stability, respectively, leading to elevated Glc_2_DAG levels. Therefore, the spontaneous mutations in the *ugtP* gene cause resistance to daptomycin. In daptomycin-resistant strains of *E. faecalis* and *S. aureus*, an increased Glc_2_DAG level has also been reported (38). Thus, based on the findings of this study, it appears that the increased Glc_2_DAG levels may be a major factor contributing to daptomycin resistance in Gram-positive bacteria. Future studies should focus on investigating the involvement of *ugtP* homologs in other bacterial species and elucidating the molecular mechanisms by which increased Glc_2_DAG levels contribute to daptomycin resistance.

## MATERIALS AND METHODS

### Strains and culture conditions

*B. subtilis* 168 *trpC2* and the mutant strains were cultured on LB agar plates or in LB liquid medium under aerobic conditions at 37°C. The *B. subtilis* gene deletion mutants carrying *ermC* were similarly cultured on LB agar plates or in LB liquid medium containing 1-µg/mL erythromycin. To overexpress UgtP, bacterial strains were cultured on LB agar plates or in LB liquid medium containing 1-mM IPTG. Details of the bacterial strains and plasmids used are listed in Table 1.

**TABLE 1:** List of bacterial strains and plasmids used

Strain or plasmid	Genotype or characteristics	Source or reference
Strains		
*Bacillus subtilis*		
168	*trpC2*	BGSC
BKE13680	*trpC2*, D*motB*::*erm*, Erm^r^	(25)
168 (BKE)	*trpC2,* originated from BKE13680	This study
BKE21910	*trpC2*, D*metA*::*erm*, Erm^r^	(25)
BKE21920	*trpC2*, D*ugtP*::*erm*, Erm^r^	(25)
BKE21910ML	*trpC2*, D*metA*::markerless	This study
*Escherichia coli*		
JM109	Host strain for cloning	Takara Bio
Plasmids		
pDR110	An integration vector, Amp^r^, Spc^r^	BGSC (25)
pDR110-metA	pDR110 with *metA*, Amp^r^, Spc^r^	This study
pDR110-ugtP	pDR110 with *ugtP*, Amp^r^, Spc^r^	This study
pDR244	Cre recombinase-expressing plasmid, Amp^r^, Spc^r^	BGSC (25)

### Screening of daptomycin-resistant mutants

The BKE library (25) was cultured in 96-well microplates at 37°C. The cultures were spotted onto agar plates containing 4 µg/mL of daptomycin using a replicator and incubated at 37°C overnight. Mutant strains that formed colonies were identified. The identified strains were re-streaked onto daptomycin-containing plates and their resistance was confirmed.

### Genetic manipulations

#### Construction of the wild-type strain

Since the colony shapes of the gene deletion mutants in the BKE library and the 168 *trpC2* strain from BGSC were different, we assumed that the genetic background differed between the parent strain of the BKE library and the 168 *trpC2* strain from BGSC. To compare the phenotypes of the BKE library mutant strains with those of the wild-type strain, we constructed a wild-type strain from the *motB*-deletion mutant (BKE13680) in the BKE library by repairing *motB*. The *motB*-deletion mutant was transformed with DNA fragments containing *motB*, cultured, and 2 µL was inoculated into the center of 0.3% LB agar plates. After overnight incubation at 37°C, the peripheral region of the colony was picked with a toothpick, and a single colony was isolated. The isolated strain was again inoculated into the center of 0.3% LB agar plates, and recovery of the swimming activity was confirmed. Correct repair of the *motB* locus was confirmed by PCR using specific oligonucleotide primers (Table 2). The colony shape was similar to that of the BKE library mutants. This strain was used as the wild-type strain in the present study.

**TABLE 2:** Primers used in this study

Primers to construct pDR110-ugtP and to confirm the *ugtP* integration	
ugtP*_*F_SalI	GTCGTCGACGCATGAAGCTTGCTTGTTGTT
ugtP*_*R_SphI	GCAGCATGCTGCGAAACGGCTTATTTTTC
Primers to construct pDR110-metA and to confirm the *metA* integration	
metA_F_HindIII	AAGAAGCTTCTGTTGTTTCCCCCTCAAC
metA_R_NheI	GCTGCTAGCTTTGCTCAGCGTATCACTAAAAA
Primers to confirm the replacement of *amyE* locus	
amyE_F	TACAGCACCGTCGATCAAAA
amyE_R	CTCGGTCCTCGTTACACCAT
Primers to construct 168 (BKE)	
motB_F	TGGACAGATCGTGCAAAGAG
motB_R	CTCGGTCCTCGTTACACCAT

#### Construction of gene deletion mutants

Genomic DNA from the *metA* or *ugtP*-deletion mutants in the BKE library was extracted using the QIAamp DNA Blood Mini Kit (Qiagen). The extracted genomic DNA was used to transform the *B. subtilis* wild-type strain, which was spread on agar plates containing erythromycin (1 µg/mL) and incubated overnight at 37°C. Erythromycin-resistant colonies were subjected to colony PCR to confirm the presence of the *metA* or *ugtP* deletions.

#### Construction of markerless *metA*-deletion mutant

The *metA*-deletion mutant was transformed with pDR244 (25) and passaged twice on agar plates containing spectinomycin (100 µg/mL) at 30°C. The colonies were then streaked onto an LB agar medium without drugs and incubated overnight at 43°C. The growing colonies were then streaked onto drug-free LB agar plates and incubated overnight at 43°C. The resulting colonies were streaked onto agar plates containing erythromycin (100 µg/mL) or spectinomycin (100 µg/mL) and confirmed to be sensitive to these antibiotics. PCR was performed to confirm the loss of erythromycin resistance markers from the *metA* gene region.

#### Construction of *ugtP*-overexpressed strain

DNA fragments containing the *ugtP* gene were amplified by PCR using oligonucleotide primers (Table 2) and genomic DNA of the wild-type strain as a template. The amplified DNA fragment was inserted into the SalI and SphI regions of pDR110 to obtain pDR110-*ugtP*. The plasmid was used to transform the *B. subtilis* wild-type strain and cultured on LB agar plates containing spectinomycin (100 µg/mL). Correct insertion of *ugtP* into the *amyE* locus was confirmed by PCR.

### Evaluation of bacterial resistance to antibiotics

To evaluate antibiotic resistance, the autoclaved LB agar medium and antibiotic solution were mixed and poured into square Petri dishes (Eiken Chemical, Tokyo, Japan). Overnight cultures of the bacterial strains were serially diluted 10-fold in 96-well microplates, and 5 µL of the diluted bacterial solution was spotted on the LB agar plates containing antibiotics and incubated at 37°C. The plates were photographed using a digital camera, and the number of colonies was counted according to a previously described method (39).

### Measurement of bacterial growth curve

Overnight cultures (2 µL) of *B. subtilis* strains were inoculated into 200 µL of LB medium containing 1-mM IPTG in a 96-well microplate and incubated at 37°C for 7 h with shaking. The OD_595_ was measured using a microplate reader (Muktiskan FC, Thermo Fisher Scientific).

### Experimental evolution of daptomycin-resistant *B. subtilis* strains

*B. subtilis* colonies were inoculated into 5 mL of LB medium supplemented with 5 µL of ethyl methanesulfonate (EMS) and cultured at 37°C overnight. The EMS-treated culture (2 µL) was inoculated into LB medium (200 µL) containing 0.125-mM calcium chloride and various concentrations of daptomycin (0.5-µg/mL increments), and incubated at 37°C without shaking overnight. Bacterial growth in the well containing the highest concentration of daptomycin was inoculated again into LB medium (200 µL) containing 0.125-mM calcium chloride and various concentrations of daptomycin. The culture process in the presence of daptomycin was repeated 15–26 times, and four resistant strains with a higher MIC than that of the wild-type strain were isolated. The *ugtP* region of the resistant strains was amplified using PCR and subjected to Sanger sequencing.

### Lipid extraction and TLC

Total lipid extraction and TLC analysis of Glc_2_DAG were performed as described previously with minor modifications (40). Overnight cultures (1 mL) of *B. subtilis* strains were inoculated in 100 mL of LB medium in a 0.5-L flask, cultured aerobically at 37°C for 4 h 30 min, after which 40 mL of the bacterial culture was centrifuged at 10,400 × *g* for 10 min at 4°C. The bacterial pellet was suspended in 1 mL of MilliQ water, and lipids were extracted using the Bligh and Dyer method (41). The lipid fraction was evaporated in a centrifugal evaporator, and the lipids were dissolved in 200 µL of chloroform:methanol (1:1 vol/vol). The samples were spotted onto a TLC silica gel 60 plate (Merck). The plate was developed in chloroform:methanol:water (65:25:4 vol/vol), sprayed with a coloring agent (10.5 mL of 15% 1-naphthol in ethanol, 6.5 mL of sulfuric acid, and 4 mL of water), and then heated at 110°C.

For phospholipid analysis, a previously described method was used with minor modifications (28, 42). Total lipids were extracted from 500 mL of the bacterial culture using the method described above. Before spotting the samples, TLC plates were immersed in 1.8% boric acid in ethanol for 2 min, dried for 15 min, and heated at 100°C for 15 min. After spotting the sample, the TLC plates were developed in chloroform:methanol:water:ammonia (56:35:2.8:1, vol/vol/vol/vol). Molybdenum blue spray reagent (Merck) was used to visualize the phospholipids.

GP-DGDAG levels were determined following a previously reported method with slight modifications (43, 44). Briefly, total lipids were extracted from 500-mL culture medium and subjected to TLC analysis as in the Glc_2_DAG detection method described above.

### DAG detection

DAG level was determined using TLC analysis following the methods described in a previous study (30). Briefly, total lipids were extracted from the 500-mL culture medium as described above, and samples were spotted onto TLC silica gel plates (Merck) developed in toluene:chloroform:acetone (7:2:1 vol/vol/vol). Afterward, the plates were visualized with a coloring agent (p-anisaldehyde 200 µL, methanol 18 mL, sulfuric acid 2 mL). The plates were heated at 100°C for 3 min to enhance the visualization of lipid spots.

### Detection of LTA

LTA levels were determined following a previous study (40) with minor modifications. Briefly, overnight cultures of *B. subtilis* were centrifuged, and the cell pellet was suspended in Tris-EDTA buffer. After lysozyme treatment at 37°C for 30 min, 3 × SDS sample buffer was added and boiled for 30 min. After centrifugation, 3 × SDS sample buffer was added to the supernatant and boiled for 10 min. The sample was then electrophoresed on a 15% SDS-polyacrylamide gel and transferred to a nitrocellulose membrane (Bio-Rad). Subsequently, the membranes were treated with 1:2,000 anti-lipoteichoic acid antibody (Hycult Biotech) for 1 h and washed three times with phosphate-buffered saline (PBS), followed by treatment with anti-mouse IgG HRP conjugate (Promega) for 1 h. After three times washing with PBS, the membrane was incubated with Western lighting Plus-ECL (PerkinElmer), and the chemiluminescent signal was detected using the iBright 1500 imaging system (Thermo Fisher Scientific).

### Evaluation of the bactericidal activity of daptomycin

The bactericidal activity of daptomycin was evaluated as described previously (45). Logarithmically growing *B. subtills* cells were centrifuged at 4000 × *g* for 5 min, and the bacterial pellet was suspended in 1 mL of PBS, diluted 10-fold, treated with 10 µg/mL of daptomycin at 37°C for 5 min, and then centrifuged at 4,000 × *g* for 5 min. The bacterial pellet was suspended in 1 mL of PBS, mixed with 1.33-µg/mL PI, and incubated at 37°C for 15 min in the dark. The percentage of bacteria that took up PI was measured by flow cytometry.

### Cytochrome C binding assay

The cytochrome c binding assay was performed as described previously (46). Overnight cultures of *B. subtills* strains were centrifuged at 2,500 × *g* for 10 min. The bacterial pellet was washed twice with buffer [20-mM 3-Morpholinopropanesulfonic acid (MOPS), 5-mM sodium citrate, and 1-mM EDTA, pH 7.0]. The cells were suspended in the same buffer and adjusted to an OD_600_ of 7. Next, 0.5-mg/mL cytochrome c was added to the cells and incubated at 37°C for 15 min. The cells were centrifuged, and the OD_530_ of the supernatant was measured. The cytochrome c concentration in the supernatant was calculated using a standard curve.

### Statistical analysis

All statistical analyses were performed using Prism software (version 9.4.1, GraphPad Software).

## SUPPLEMENTAL MATERIAL

Supplemental material jb.00307-24-s0001.pdf Fig. S1 and S2.

ASM does not own the copyrights to Supplemental Material that may be linked to, or accessed through, an article. The authors have granted ASM a non-exclusive, world-wide license to publish the Supplemental Material files. Please contact the corresponding author directly for reuse.
