# Supplementary material for: Overexpression of diglucosyldiacylglycerol synthase leads to daptomycin resistance in Bacillus subtilis
Source: J Bacteriol. 2024 Sep 5;206(10):e00307-24. doi: 10.1128/jb.00307-24 (PMC11500525; doi:10.1128/jb.00307-24)

Fig. S1. Growth curves of the wild-type, Δ*metA*, and *ugtP*-overexpressed strains

Growth curves of the wild-type, Δ*metA*, vector-transformed, and *ugtP*-overexpressed strains were examined. Data are presented as means ± standard error (n = 3).

Fig. S2. Daptomycin resistance of Δ*metA* is not canceled by *metA* complementation

Overnight cultures of the wild-type strain (WT), the *metA*-deletion mutant (Δ*metA*), and the *metA*-complementation strain (Δ*metA*/*spec*-P_{spank}-*metA*) were serially diluted 10-fold, spotted onto Luria-Bertani agar plates containing 1 mM isopropyl-beta-D-thiogalactopyranosid supplemented with or without daptomycin 4 μg/mL, and incubated at 37°C overnight.

Figure S1

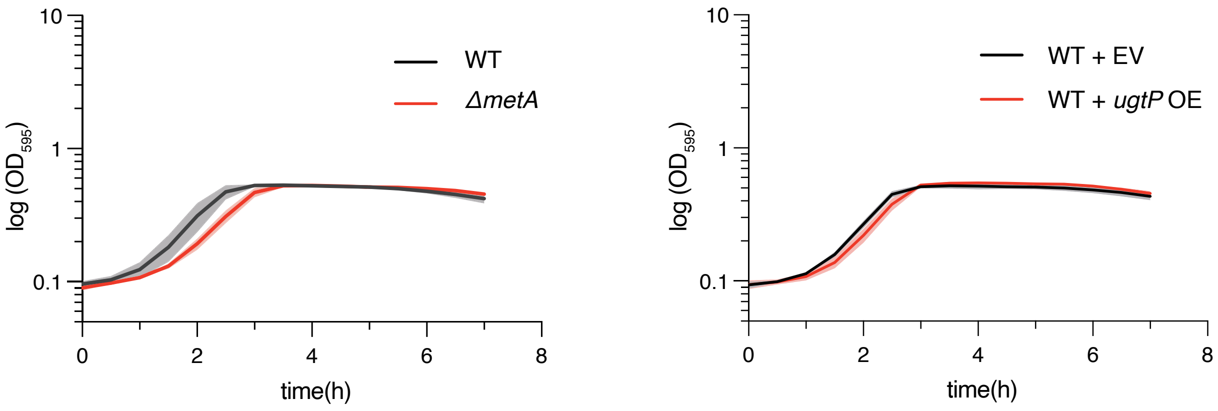

Figure S2

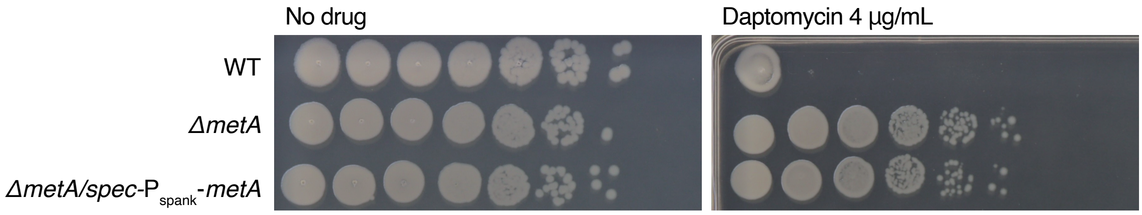

Supplement: Supplemental material — Fig. S1 and S2. [file jb.00307-24-s0001.pdf]
